# Modelling of oedemous limbs and venous ulcers using partial differential equations

**DOI:** 10.1186/1742-4682-2-28

**Published:** 2005-08-03

**Authors:** Hassan Ugail, Michael J Wilson

**Affiliations:** 1 School of Informatics, University of Bradford, Bradford BD7 1DP, UK; 2 Department of Applied Mathematics, University of Leeds, Leeds LS2 9JT, UK

## Abstract

**Background:**

Oedema, commonly known as tissue swelling, occurs mainly on the leg and the arm. The condition may be associated with a range of causes such as venous diseases, trauma, infection, joint disease and orthopaedic surgery. Oedema is caused by both lymphatic and chronic venous insufficiency, which leads to pooling of blood and fluid in the extremities. This results in swelling, mild redness and scaling of the skin, all of which can culminate in ulceration.

**Methods:**

We present a method to model a wide variety of geometries of limbs affected by oedema and venous ulcers. The shape modelling is based on the PDE method where a set of boundary curves are extracted from 3D scan data and are utilised as boundary conditions to solve a PDE, which provides the geometry of an affected limb. For this work we utilise a mixture of fourth order and sixth order PDEs, the solutions of which enable us to obtain a good representative shape of the limb and associated ulcers in question.

**Results:**

A series of examples are discussed demonstrating the capability of the method to produce good representative shapes of limbs by utilising a series of curves extracted from the scan data. In particular we show how the method could be used to model the shape of an arm and a leg with an associated ulcer.

**Conclusion:**

We show how PDE based shape modelling techniques can be utilised to generate a variety of limb shapes and associated ulcers by means of a series of curves extracted from scan data. We also discuss how the method could be used to manipulate a generic shape of a limb and an associated wound so that the model could be fine-tuned for a particular patient.

## 1 Introduction

**Figure 1:**
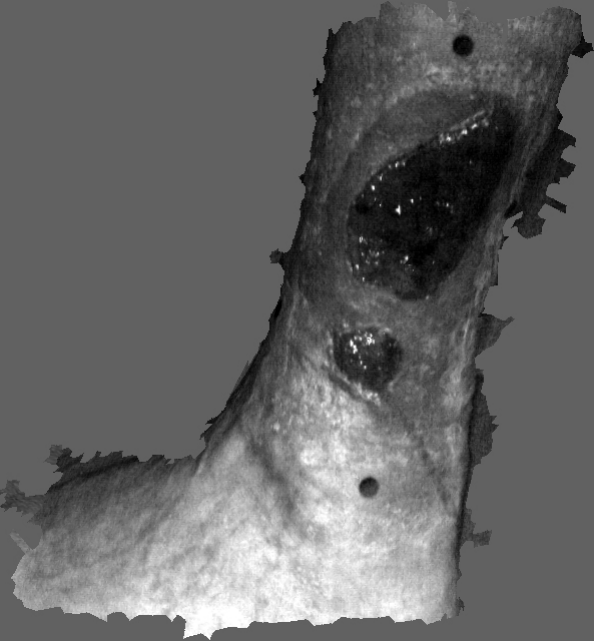
An example of an oedemous leg infected with oedema venous ulcers.

Oedema, commonly known as tissue swelling, is associated with a range of causes such as venous disease, trauma, infection, joint disease, orthopaedic surgery and removal of the lymph nodes. Oedema and associated venous ulcers occur on mainly on the leg and the arm. It can be a painful, embarrassing and costly disorder [1,2]. It occurs widely in the general population, especially from late middle age, in diabetics and in immobile patients [3-5]. Apart from the tissue swellings the ulcers themselves can typically range in size from around 0.5 cm to 10 cm across, and are of variable depth [6,7]. Fig. 1 shows an example of an oedemous leg infected with venous ulcers.

An important task, during the treatment of oedema and venous ulcers, is the measurement of the amount of oedema as well as the area and volume of the ulcer wounds. This is because without an accurate and objective means of measuring changes in the size or shape of ulcers, it is difficult or impossible to evaluate the efficiency of the available therapies properly. Therefore, a prerequisite for this development is a reliable method of measuring ulcers. There exist a variety of measurement methods none of which is ideal. At present direct contacting measurements are widely used but they are not accurate, carry a risk of infection and are, to say the least, uncomfortable for the patient. For example, conventional techniques for measuring the area and volume of wounds depend on making physical contact with the wound, for example by drawing around the periphery on an acetate sheet or by making an alginate cast of the wound [6]. There is currently significant interest in developing non-invasive measurement systems using optical methods such as 'structured light' (a technique that projects stripes on to a surface and infers the shape from changes in the linearity of the reflected stripe) [8] or stereo-photogrammetry. The availability of high-resolution 3D digital cameras, increasing computing power and the development of software techniques for manipulating three-dimensional information have benefited this area. However, equipment associated with these sorts of measurement methods is not often portable and is often costly, thus making it prohibitive for routine medical use.

The aim of this paper is to show how it is possible to develop a system for measuring the shape and size of limbs and venous ulcers by means of utilising an economical mathematical model. In particular, one of the outcomes we hope to achieve from this work is a technique with potential for clinical use. For this, a small number of key measurements of limbs (with minimal possible contact with the limb and the associated ulcer), made using readily available instruments such as callipers and tape measures, can be input to a computer program. The program will then be able to reconstruct a good estimate of the limb shape and dimensions. It is believed that such a technique will provide a cheap, efficient, non-invasive instrument for measuring the degree of oedema and consequently enabling various treatment plans to be evaluated.

At present there exists a wide variety of methods that can be utilised to generate the geometry of limbs affected by oedema and venous ulcers. These include boundary based methods such as polygon based design [9], extrusions and surface of revolution [10] and polynomial patches [11]; procedural modelling such as implicit surfaces [12] and fractals [13]; and volumetric models such as constructive solid geometry [14] and subdivision [15]. Many of these techniques, especially polygon based design and polynomial patches, would be very appropriate for limb shape reconstruction, although they may not be ideally suited for the problem we address here. For example, conventional spline patches would require a large array of control points and weights in order to represent a realistic shape of a limb and associated wounds.

In this initial stage of the work we are concerned with developing efficient techniques in order to perform two important tasks. They are: the generation of smooth surfaces resembling the surface data obtained from a 3D scanner; and, once a smooth surface is obtained, manipulation of the geometry so as to obtain a good representation of the limb shape for any given patient. To do this we utilise real data from a series of surface scans provided by a medical partner namely, the Department of Medical Physics and Vascular Surgery of Bradford Teaching Hospitals National Health Services Trust (BTHNHST), UK, with whom we work closely on these problems. The department of Medical Physics at BTHNHST acquired the surface data using multiple-camera photogrammetry with a DSP400 system from 3dMD Ltd. This commercial technology has been widely used for acquiring medical images, especially in the USA, and captures data in a few milliseconds. The surface resolution (i.e. the separation of data points) is approximately 2 mm with a positional accuracy of approximately 0.2 mm. When developing our PDE based techniques for modelling human limbs, which are affected by oedema and venous ulcers, our medical partner has two aims. Firstly they require a compact and smooth surface representation of their captured data. Secondly, and rather more importantly, they require a modelling tool that would enable them to manipulate the shape of a given limb so as to provide a good representative limb shape of any given patient.

In this paper we utilise the so called PDE method [16-18] to address the problem. A positive feature of the PDE method is that it can define surfaces in terms of a small set of design variables [19], instead of many hundreds of control points. In broad terms this is because its boundary-value approach means that PDE surfaces are defined by data distributed around just their boundaries, instead of data distributed over their surface area, e.g. control points. Thus, a PDE model, when changed by altering the values of its design parameters, remains continuous; there is no need for a designer to intervene in order to close up any holes that might appear at patch boundaries. In the present context, this means that PDE surfaces can be made to adapt to changes in the shape of the limb and the associated wounds.

## 2 PDE Surfaces

A PDE surface is a parametric surface patch , defined as a function of two parameters *u *and *v *on a finite domain Ω (⊂) *R*^2 ^by regarding the function  as a mapping of a point in Ω to a point  in the physical space. The shape of the surface patch is usually determined by specifying a set of boundary data at the edge of (∂)Ω. Typically the boundary data are specified in the form of  and a number of its derivatives on (∂)Ω. Hence, by casting the surface generation as a boundary value problem, the surface  is regarded as a solution of an elliptic PDE.

Various elliptic PDEs could be used; the ones we utilise for this work are based on the biharmonic and triharmonic equations, namely,



and



Also, periodic boundary conditions are very often considered. Assuming we are working with the above two elliptic PDEs, we require them to satisfy a set of 2*N *conditions, where *N *is 2 in the case of Equation (1) and *N *3 in the case of Equation (2). The general form of these conditions can then be written as,

**X**(0, *v*) = **f**_1_(*v*),     (3)

**X**(*u*_*i*_, *v*) = **g**_*i*_(*v*), *i *= 2 ... 2*N *- 1     (4)

**X**(1, *v*) = **f**_2*N*_(*v*),     (5)

where **f**_1_(*v*) in Equation (3) and **f**_2*N*_(*v*) in Equation (5) are function conditions specified at *u *= 0 and *u *= 1 respectively. The conditions **X**(*u*_*i*_, *v*) = **g**_*i*_(*v*) in Equation (4) can take the form either

**X**(*u*_*i*_, *v*) = **f**_*i *_for 0 <*u*_*i *_< 1, *i *= 2 ... 2*N *- 1,     (6)

or



In simpler terms the above conditions imply that for a PDE surface patch of order 2*N*, we can specify two function conditions, as given in Equations (3) and (5), that should be satisfied at the edges (at *u *= 0 and *u *= 1) of the surface patch, and a number of function or derivative conditions, as given in Equation (4), amounting to 2*N *– 2 conditions that the PDE should also satisfy.

### 2.1 Solution of the PDEs

There exist many methods for solving Equations (1) and (2) ranging from analytic solutions to sophisticated numerical methods. The problems we address in this paper involve modelling of human limbs, which are essentially closed and cylindrical, and therefore the broad range of shapes encountered can be incorporated by solving the chosen PDEs with periodic conditions. Note here periodic conditions imply that for the *v *parameter the condition, , is satisfied. Thus, for the work described here, we restrict ourselves to periodic conditions and obtain a closed form analytic solution of Equations (1) and (2).

Choosing the parametric region to be 0 ≤ *u *≤ 1 and 0 ≤ *v *≤ 2π, and assuming that the conditions given in Equations (3), (4) and (5) are periodic functions, we can use the method of separation of variables and spectral approximation [20] to write down the analytic solution of Equations (1) and (2) as,



where  is a polynomial function and  and  are exponential functions. The specific forms of  and  for the case of Equations (1) can be found in [17] and for the case of Equations (2) can be found in [21].

The main point to bear in mind regarding the above solution method is that it enables one to represent a set of general periodic conditions in terms of a finite *M *Fourier series, where *M *is typically taken to be ≤ 10, whilst the term , which acts as a correction term, enables the conditions to be satisfied exactly. Detailed discussions of this solution method can be found in [20].

### 2.2 Methods of Generating PDE Surfaces

In this section we discuss a series of examples, showing the various methods by which PDE surfaces can be generated where the PDEs are chosen to be Equations (1) and (2) and the conditions are taken in the format described in Equations (3), (4) and (5).

**Figure 2:**
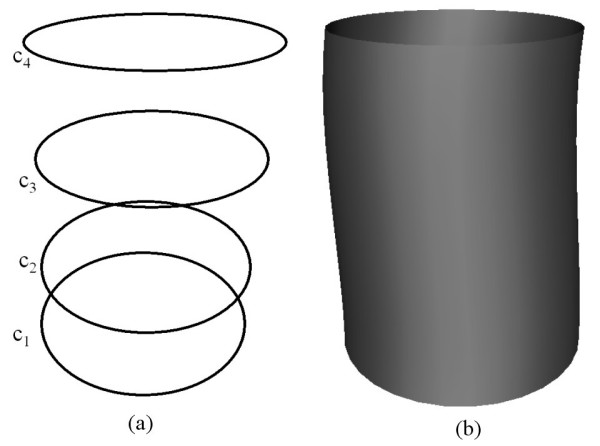
The shape of a surface generated by the fourth order **PDE **where the conditions are all taken to be function conditions (**a**) The conditions defined in the form of curves in 3-space. (**b**) The resulting surface shape.

As a first example we show how a fourth order PDE surface is generated where all the conditions are taken to be function conditions. Fig. 2(b) shows the shape of a surface generated by the fourth order PDE where the conditions are specified in terms of the curves shown in Fig. 2(a). In particular, the conditions are such that:  and . Since we are taking four function conditions to solve the fourth order PDE, all the curves in this case lie on the resulting surface. Thus, in this particular case the resulting PDE surface is a smooth interpolation between the given set of functional conditions.

**Figure 4:**
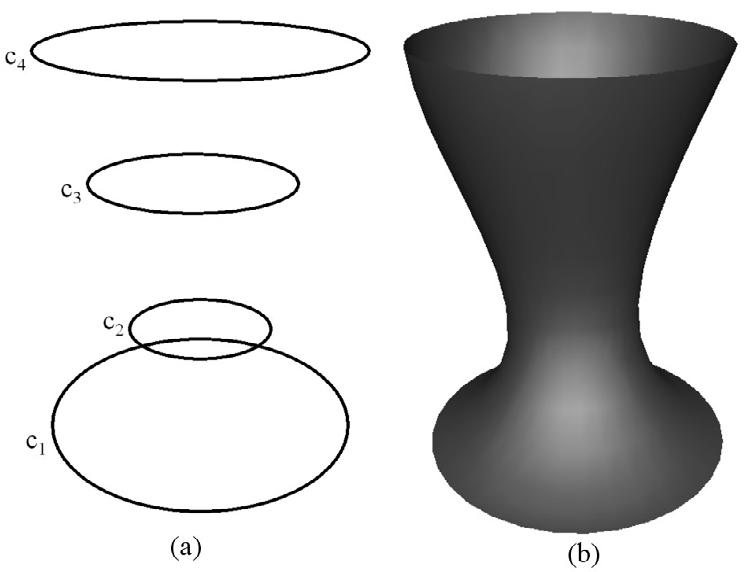
The shape of a surface generated by the fourth order **PDE **where the boundary conditions are taken to be both positions and derivatives (**a**) The boundary conditions defined in the form of curves in 3-space. (**b**) The resulting surface shape.

The next example shows how a fourth order PDE surface is generated when the conditions are taken to be a mixture of function conditions and derivative conditions. Fig. 4(b) shows the shape of a surface generated by the fourth order PDE where two function boundary conditions and two derivative boundary conditions are specified in terms of the curves shown in Fig. 4(a). In particular, the boundary conditions are chosen such that:  and , where *s *is a scalar. In this case the surface patch generated as a solution to the fourth order PDE contains the boundary curves *c*_1 _and *c*_4 _whilst it does not necessarily contain the curves *c*_2 _and *c*_3_. A typical scenario where a surface of this nature is required would be a blend design where the derivative boundary curves can be adjusted to produce a smooth blend surface that bridges between two primary surfaces.

**Figure 3:**
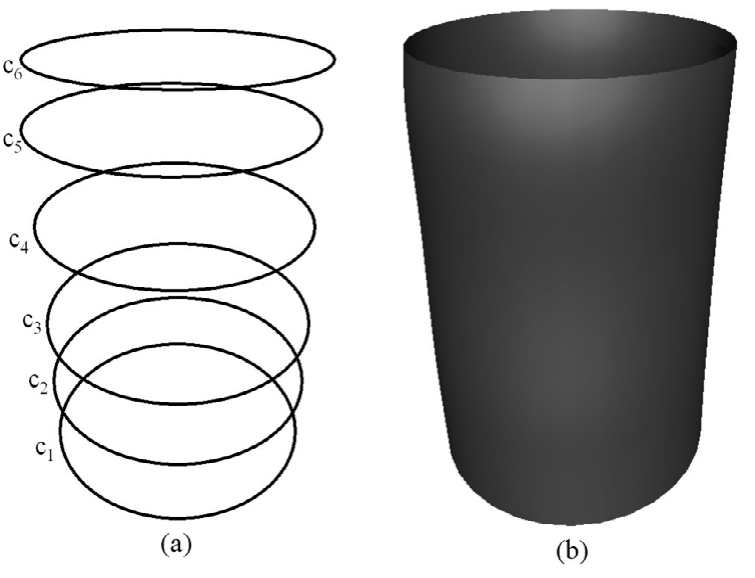
The shape of a surface generated by the sixth order **PDE **where the conditions are all taken to be positions (**a**) The conditions defined in the form of curves in 3-space. (**b**) The resulting surface shape.

Fig. 3(b) shows the shape of a surface generated by the sixth order PDE where the conditions are all taken to be positions specified in terms of the curves shown in Fig. 3(a). In particular, the boundary conditions are such that  and . As in the first example of the fourth order case, since we are taking six function conditions to solve the sixth order PDE, all the curves in this case lie on the resulting surface. Thus, the resulting PDE surface is a smooth interpolation between the six prescribed curves.

**Figure 5:**
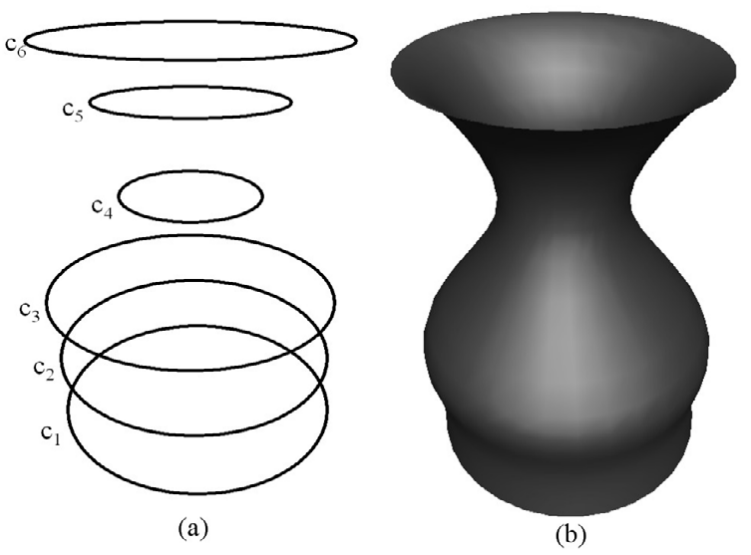
The shape of a surface generated by the sixth order **PDE **where the boundary conditions are taken to be both positions and derivatives (**a**) The boundary conditions defined in the form of curves in 3-space. (**b**) The resulting surface shape.

As a final example we show how a sixth order PDE surface is generated where the boundary conditions are taken to be a mixture of function boundary conditions and derivative conditions (both first and second order). Fig. 5(b) shows the shape of a surface generated by the sixth order PDE where two function boundary conditions, two first order derivative boundary conditions and two second order derivative boundary conditions are specified in terms of the curves shown in Fig. 5(a). In particular, the boundary conditions are chosen such that  and . where *s *and *t *are scalars. As in the example of fourth order case shown in Fig. 4 the surface generated in this case contains the curves *c*_1 _and *c*_6 _whilst it does not necessarily contain the rest of the curves. Again this type of surface shape can be utilised in blend design where higher order continuity is desired in producing a smooth blend surface that bridges between two primary surfaces. As one can see from the format of these derivative condition definitions, the derivative conditions are all defined using simple finite difference schemes. The curves defining the derivative conditions provide an intuitive shape manipulation tool in that the shape of the surface closely follows the shape of the boundary conditions.

The above examples demonstrate how PDE surfaces of order four and six can be utilised to generate surface shapes, which are applicable to a wide variety of design scenarios. Thus, the basic idea here is to generate a series of curves (both function and derivative) that can be utilised to define the boundary conditions for the chosen PDE. As seen in the examples, the resulting surface shape can always be intuitively predicted from the shapes of the chosen curves.

## 3 Modelling of Limbs and Ulcers

In this section we discuss the shape modelling of human limbs affected by oedema and venous ulcers. In what follows, we discuss two examples of shape modelling of human limbs namely modelling of an arm shape and modelling of a leg shape with an ulcer. We utilise a mixture of PDEs of order four and six in order to model the surface shapes in question. In order to generate a representative smooth PDE surface shape, we extract a series of curves along the profile of the geometric model.

**Figure 6:**
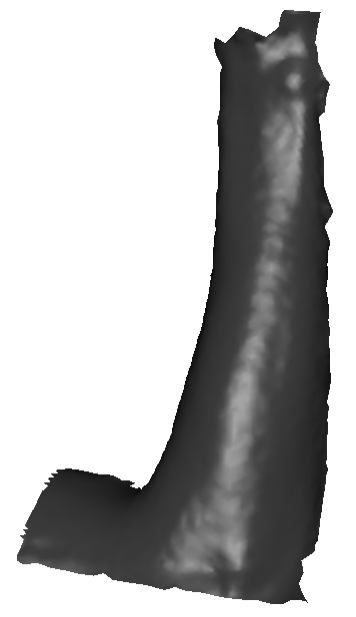
Scanned surface data of an arm.

Fig. 6 shows a typical surface scan data set provided by the medical partner where in this particular case the data set corresponds to an arm shape. Note that scan data are only available for half the surface. In order to generate a representative PDE surface shape, we extract a series of curves along the profile of the geometric model. To do this, first we import the geometric model into an interactive graphical environment through which we can examine and interact with the model. The geometric definitions of the scan data are provided in .obj file format where the 3D polygonal data with connectivity information are readily available. This enables us to display the model as well as compute the normal curvature distribution across the surface. A series of regions on the scan data model are then manually identified based on changes in the surface curvature. These regions are then utilised to determine the number of surface patches required to produce a good representative model of the limb in question. In determining the number of PDE surface patches required the aim is to reduce the number of patches that need to be utilised to produce a good representative geometric model with given accuracy. Once the number of surface patches required is decided the appropriate number of curves for each surface patch is extracted from the scan geometry data. To do this we create a series of free-form cubic spline curves within the interactive environment. The spline curves are then projected on to the scan geometry at the positions where the PDE curves are to be extracted. Note that the surface data obtained in this case do not naturally give us curves that are periodic. Thus, in this case, for each curve extracted, a series of fictitious points is added to each curve in order to make the curve periodic. The surfaces are then generated using the analytic solution described previously, where the surface is generated for the region 0 ≤ *v *≤ π which forms the portion of the curves extracted from the scanned data.

### 3.1 Example 1: Modelling of an Arm Shape

**Figure 7:**
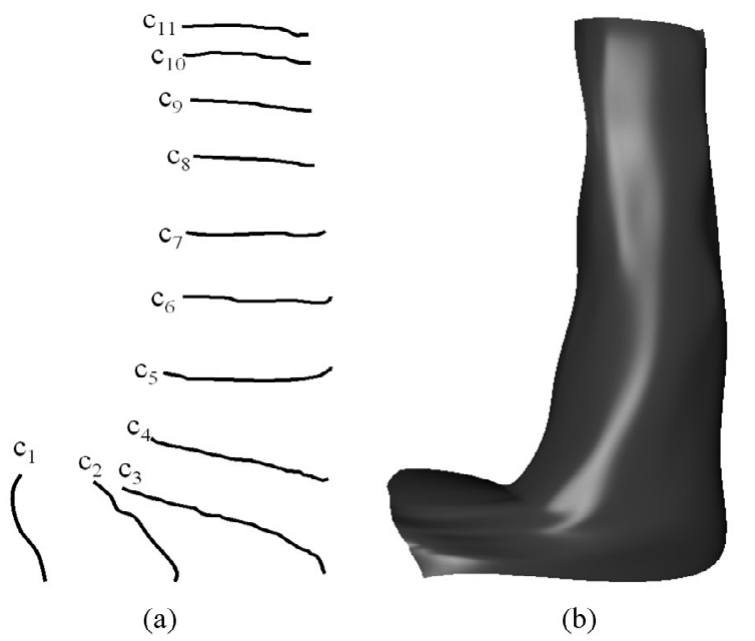
The arm shape generated using **PDE **surfaces by means of utilising curves extracted from scanned data (**a**) The extracted curves. (**b**) The resulting surface shape generated using two fourth order patches and a sixth order patch.

As a first example, we discuss the modelling of the shape of a human arm. Fig. 6 shows the scan surface data corresponding to an arm shape provided by the medical partner. Fig. 7(a) shows a series of curves extracted from the original scan surface data. Fig. 7(b) shows the arm shape generated using PDE surfaces. In particular the shape is generated as a combination of two fourth order patches and a single sixth order surface patch. i.e. the curves *c*_5_, *c*_6_, *c*_7 _and *c*_8 _and *c*_8_, *c*_9_, *c*_10 _and *c*_11 _form boundary conditions for two fourth order surface patches with the common boundary at *c*_8 _whereby all the conditions are taken to be position conditions. The curves *c*_1_, *c*_2_, *c*_3 _and *c*_5 _form a sixth order surface patch where *c*_1_, *c*_2_, *c*_4_, *c*_5 _are taken to be four position conditions and the differences between *c*_2_, *c*_3 _and *c*_5_, *c*_4 _are taken to be two first order derivative boundary conditions. The value of the parameter *s *is taken to be 0.34.

### 3.2 Example 2: Modelling of a Leg Shape with a Venous Ulcer

**Figure 8:**
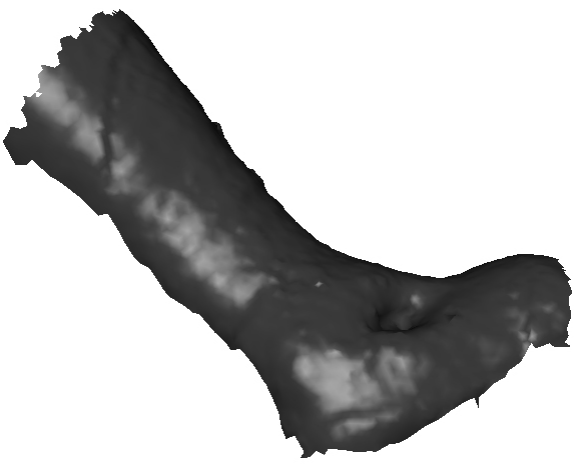
Scanned surface data of a leg infected with an ulcer.

**Figure 9:**
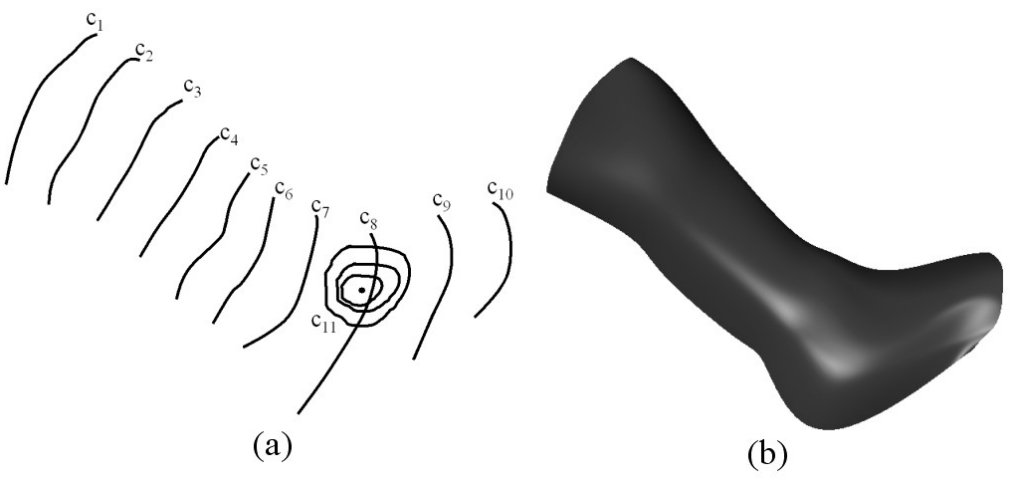
The main leg shape generated using **PDE **surfaces by means of utilising curves extracted from scan data (**a**) The extracted curves (including the wound). (**b**) The resulting surface shape corresponding to the main shape of the leg, generated using two sixth order patches and a sixth order patches.

As a second example we discuss the modelling of the shape of a leg infected with a venous ulcer. Fig. 8 shows the scan surface data, corresponding to the infected leg with a venous ulcer. As in the previous example, in order to generate a representative smooth surface shape, we first extract a series of curves along the leg and the associated wound. Fig. 9(a) shows a series of curves extracted from the original scan data.

In order to create a smooth shape that closely resembles the geometry of the leg, we utilise two sixth order patches to generate the main portion of the leg. Thus, the curves *c*_1_, *c*_2_, *c*_3_, *c*_4_, *c*_5 _and *c*_6 _form the position condition for a sixth order surface patch where the surface patch passes through these curves. The other surface patch is generated using the curves *c*_6_, *c*_7_, *c*_8_, *c*_9 _and *c*_10 _where the curve *c*_6 _is common to both surface patches. Moreover, for the later surface patch the curves *c*_6_, *c*_7 _and *c*_9_, *c*_10 _form four position boundary conditions and the differences between the curves *c*_7_, *c*_8 _and *c*_9_, *c*_8 _form two first order derivative boundary condition thus ensuring a smooth geometry transition between the foot and the leg. The parameter *s *is taken to be 0.12.

**Figure 10:**
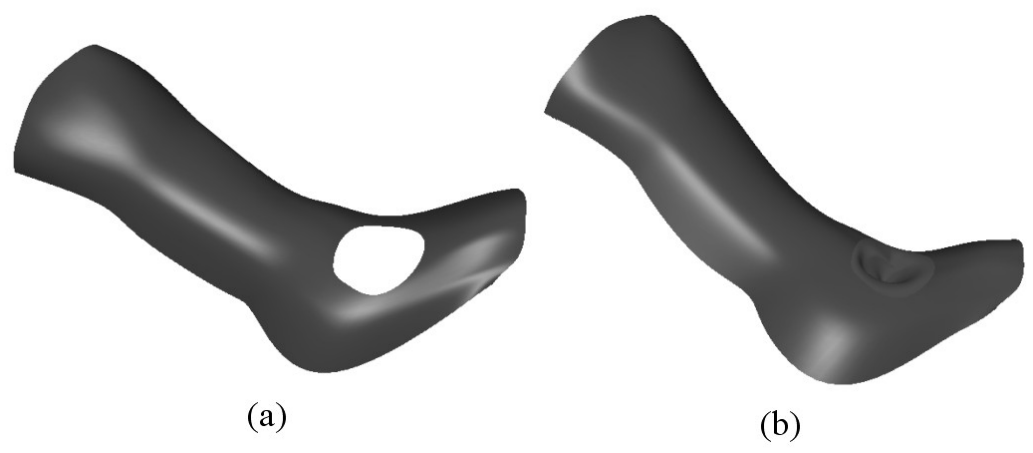
Leg and ulcer geometry. (**a**) A portion of the surface is trimmed out using a curve resembling the edge of the ulcer wound (**b**) The complete leg geometry with the ulcer wound.

To generate the wound shape on the leg, we define a curve on the PDE leg surface that closely resembles the edge of the wound. This curve, marked as *c*_11 _as shown in Fig. 9(a), is generated using the (*u*, *v*) parameter space of the corresponding the PDE surface. Next the surface portion corresponding to the interior of the curve *c*_11 _is trimmed out. This trimming process is again carried out using the (*u*, *v*) parameter space as described in [17]. Fig. 10(a) shows the main leg surface with the trim.

Once the appropriate trimming is carried out, a separate fourth order patch resembling the shape of the wound is generated where the curve *c*_11 _which lies on the main leg surface is utilised as one of the four position boundary conditions. Fig. 10(b) shows the complete leg shape along with the ulcer wound.

Both the examples discussed above show how PDE surfaces of low order (i.e. order 4 and 6 in this case) can be utilised to generate good representative shapes using little information from the scan data. One could argue that a single PDE surface of higher order can be equally well suited to generating a single surface patch through a given number of curves. However, from the min-max principle for elliptic PDEs it is well known that PDEs of higher order (i.e. orders above 6) are difficult to control. Choosing lower order PDEs to generate the surface therefore makes sense. It is also noteworthy that the parameters *s *and *t *and the difference between the corresponding position and derivative curves enable both the size and the direction of the derivative boundary conditions at the edge of a given surface patch to be controlled. The derivative boundary conditions are used to control the smoothness of the blend between two surface patches. Such a tool cannot be deployed to reduce the number of curves used and hence the number of surface patches utilised to model the complete limb.

## 4 Conclusion

This paper describes how the PDE method can be utilised to model a wide variety of geometries of limbs affected by oedema and venous ulcers. The shape modelling is based on solving a PDE subject to a set of curves extracted from 3D scan data providing the shape of the affected limbs. For this work we utilise a mixture of fourth order and sixth order PDEs, depending on the accuracy and continuity requirements for obtaining a good representative shape of the limb and associated ulcers in question.

In this work we are concerned with developing efficient techniques in order to undertake two important tasks. They are: the generation of smooth surfaces closely resembling the surface data obtained from a 3D scanner; and once a smooth surface is obtained, manipulation of the geometry so as to provide a good representative limb geometry shape for any given patient. Thus, the prime aim of the technique we discuss here is to generate a good representative shape of the limb quickly from the scanned data and to be able to manipulate that shape efficiently. It is noteworthy that the process of PDE geometry generation from the scan data is currently carried out manually. We are currently working on developing a methodology for automating this process. We have shown examples that clearly demonstrate the ability of PDE shape modelling techniques to generate a variety of limb shapes and associated venous ulcers. The geometry models themselves are flexible in terms of their manipulation capabilities, i.e. the manipulation of geometry can be carried out via the manipulation of curves defining the surface.

Our future direction in this work is to define a shape parameterisation tool for limbs where a set of shape parameters can be associated with the curves. Such shape parameterisation can then be utilised to fine tune a given generic limb model to suit to a handful of data measured from a given patient's limb. This will enable one to develop efficient non-invasive techniques for measuring various properties (such as surface area and volume) of oedema and venous ulcers.
